# Association between serum/plasma levels of adiponectin and obstructive sleep apnea hypopnea syndrome: a meta-analysis

**DOI:** 10.1186/s12944-019-0973-z

**Published:** 2019-01-26

**Authors:** Mi Lu, Fang Fang, Zhenjia Wang, Peng Wei, Chunhua Hu, Yongxiang Wei

**Affiliations:** 1 The Key Laboratory of Upper Airway Dysfunction-Related Cardiovascular Diseases, Beijing Institute of Heart, Lung and Blood Vessel Diseases, No. 2 Anzhen Road, Beijing, 100029 China; 2 Department of Sleep Medical Center, Beijing Anzhen Hospital, Capital Medical University, No. 2 Anzhen Road, Beijing, 100029 China; 3 Department of Otolaryngology Head and Neck Surgery, Beijing Anzhen Hospital, Capital Medical University, Beijing Institute of Heart, Lung and Blood Vessel Diseases, No. 2 Anzhen Road, Beijing, 100029 China; 4 Department of Radiology, Beijing Anzhen Hospital, Capital Medical University, No. 2 Anzhen Road, Beijing, 100029 China

**Keywords:** Obstructive sleep apnea hypopnea syndrome (OSAHS), Adiponectin, Meta-analysis

## Abstract

**Background:**

The relationship between obstructive sleep apnea hypopnea syndrome (OSAHS) and a variety of disease from obesity, type 2 diabetes mellitus and cardiovascular disease has been investigated previously. Reduced adiponectin levels are also associated with increased risk of these disease. However, whether serum/plasma adiponectin levels in OSAHS patients are lower than their counterparts remain controversial. Therefore, this study evaluated the association between serum/plasma adiponectin levels and OSAHS.

**Methods:**

We performed a comprehensive literature search to locate eligible articles published on electronic databases including PubMed, EMBASE, Cochrane Library, WANFANG (Chinese database), VIP (Chinese Database) and Chinese National Knowledge Infrastructure (CNKI). The methodological quality of included studies was evaluated using the Newcastle-Ottawa scale (NOS). Pooled standard mean difference (SMD) with 95% confidence interval (CI) was calculated as effect size. Heterogeneity test was performed by Cochrane Q test and I^2^ test. Subgroup analysis and meta-regression analysis were employed to detect the sources of the heterogeneity. RevMan 5.3 and Stata 12.0 software were used in this meta-analysis for data synthesis.

**Results:**

A total of 20 eligible studies with 28 databases involving 1356 participants were included in this meta-analysis. Results revealed that serum/plasma adiponectin levels in OSAHS patients were significantly lower than that in controls [SMD = − 0.71, 95% CI = − 0.92 to − 0.49, *p* < 0.001]. Subgroup analysis indicated that the heterogeneity would decreased when subgroup analysis was stratified by race. In addition, meta-regression analysis also suggested that the adiponectin levels were only significantly correlated with race. The removal of any independent study did not affect the pooled SMD in the sensitivity analysis.

**Conclusion:**

The serum/plasma adiponectin levels were significantly lower in OSAHS patients than that in control subjects, suggesting a possible role of adiponectin in OSAHS pathogenesis, deserves further studies as a potential marker of OSAHS.

## Introduction

Obstructive sleep apnea hypopnea syndrome (OSAHS), the most frequent sleep-related breathing disorder, is characterized by repetitive events of partial or complete collapse of upper airway during sleep. Clinically, it is characterized by snoring, witnessed apneic episodes, marked sleep fragmentation and daytime sleepiness, which would lead to metabolic disturbance, impaired quality of life, high morbidity as well as high mortality [1]. Approximately 10% of middle-aged men and 3% of middle-aged women are estimated to have moderate-severe OSAHS in the developed world [2]. The pathogenesis responsible for the syndrome is not completely elucidated, but there exist multiple potential etiologies. One of the most important risk factors is obesity, especially visceral obesity [3, 4]. In obese population, the prevalence of OSAHS reaches up to 40–50% [5]. In addition, OSAHS is associated with insulin resistance which can account for a further increase in metabolic syndrome especially of type 2 diabetes mellitus and cardio-cerebrovascular diseases [6].

Adiponectin (Acrp30) is one of the common adipocytokines largely secreted by adipocytes and has insulin-sensitizing, anti-inflammatory and anti-atherosclerosis properties [7]. Reduced levels of adiponectin are commonly observed in a variety of states associated with obesity and insulin resistance, such as type 2 diabetes mellitus. However, the relationship between OSAHS and serum/plasma adiponectin levels is complex and multidirectional. The complex relationship is due in part to the fact that obesity could be a cause, consequence, or confounding factor of OSAHS. In addition, some studies revealed that adiponectin levels in OSAHS patients were lower than that in non-OSAHS group [8–10]. While Wolk et al. found that higher adiponectin levels in OSAHS patients compared to controls [11]. Tokuda et al. and Ursavas et al. revealed that there was no significant difference of adiponectin levels in OSAHS patients when compared to controls [12, 13].

Thus, we sought to perform a meta-analysis using all available relevant studies to assess the association between adiponectin levels and OSAHS. Given that most of the previous findings were confounded by obesity, sex and age, we only included studies that found no statistically significant difference between OSAHS patients and controls in terms of age, gender and body mass index (BMI) to address these possible confounding factors.

## Methods

This meta-analysis is being reported in accordance with Preferred Reporting items for Systematic Reviews and Meta-analysis (PRISMA) statement [14].

### Search strategy

We performed a comprehensive literature search to locate eligible articles published on electronic databases including PubMed, EMBASE, Cochrane Library, WANFANG (Chinese database), VIP (Chinese Database) and Chinese National Knowledge Infrastructure (CNKI). Keywords and search strategy were as follows: “obstructive sleep apnea hypopnea syndrome” or “OSAHS” or “obstructive sleep apnea” or “OSA” or “obstructive sleep apnea syndrome” or “OSAS” or “obstructive sleep hypopnea” or “sleep apnea” combined with “adiponectin” or “ADPN” or “APN”. The electronic databases were searched from inception through January 2018. Besides, the references cited in these articles were reviewed to identify additional publications. We only recruited data from fully published articles written in English and Chinese.

### Study selection

Two reviewers first independently reviewed the titles and abstracts to identify relevant articles. A second screening was based on full-text articles to further see whether they were eligible for inclusion. Any disagreement was resolved by discussion.

### Inclusion and exclusion criteria of literature

The studies that satisfied the following criteria were included:

1. The study design was a case-control study that must have reported values in mean and standard deviation or median with range of adiponectin levels;
2. The study must have included at least two separate groups with one being a group

consisting of individuals with OSAHS and the other consisting of individuals without OSAHS;

3) OSAHS was defined as apnea hypopnea index (AHI) ≥ 5;
4) All OSAHS patients were diagnosed for the first time, without receiving any form of treatment;
5) No statistically significant difference was found between OSAHS patients and controls in terms of age, gender and BMI;
6) All participants were adults (age > 18 years).

The exclusion criteria were:

1. Conference abstracts, reviews articles and case reports;
2. Original papers that did not contain precise data about serum/plasma levels of adiponectin in patients or controls;
3. Studies were not performed in humans;
4. Duplicate publication of articles.

### Data extraction

The following information was recorded from each included study: first author’s name, publication year, population country, total simple size, serum/plasma adiponectin levels in patients or controls, age, gender, BMI and assay approaches for adiponectin levels.

### Quality assessment

The methodological quality of included studies was evaluated using the Newcastle-Ottawa scale (NOS) by two investigators independently. Discord was resolved by a third reviewer. The quality scale consists of three parts: selection, comparability and exposure assessment. The quality score ranges from 0 to 9. In our meta-analysis, we considered a study which is equal to or higher than 6 stars as a high-quality study.

### Statistical analysis

Due to the inconsistency of measurement units and assay approaches, standardized mean difference (SMD) with 95% confidence intervals (CI) was chosen as effect size. Heterogeneity test was performed by Cochrane Q test and I^2^ test. Generally, if *P* < 0.05 (Q-test) or I^2^ > 50%, the heterogeneity was thought to exist and then the random-effect models would be used. Otherwise, fixed-effect models would be applied. An I^2^ of 25 to 49% was considered to represent a low level of heterogeneity, 50 to 74% a moderate level, and 75 to 100% a high level. Subgroup analysis was performed to assess the impact of race, adiponectin source, assay approaches, average age, BMI and AHI. Sensitivity analysis was conducted to evaluate the stability of pooled results. Potential publication bias was assessed by using the funnel plots, Begg’s rank correlation method and the Egger’s linear regression method. The statistical analysis was performed with Revman 5.3 and Stata12.0 software. *P* < 0.05 were considered statistically significant.

## Results

### Search result

**Fig. 1:**
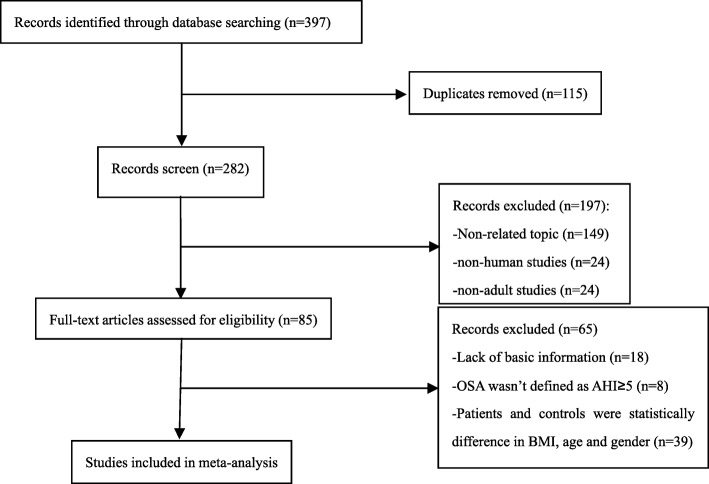
Flow diagram of screened and included papers

A total of 397 relevant articles were preliminarily identified. After removing duplicates and screening by titles and abstracts, 312 articles were excluded. The remaining 85 articles were projected to be assessed according to the inclusion and exclusion criteria after reading the full-text. Then another 65 articles were excluded due to different reasons. Finally, a total of 20 studies with 28 datasets met inclusion criteria and were pooled for this meta-analysis [8, 10–12, 15–30]. A flow diagram of the study selection process is presented in Fig. 1.

### Characteristics of the eligible studies

A total of 20 studies involving 1356 participants (OSAHS subjects [*N* = 878] and controls [*N* = 478]) were included in our meta-analysis. The information of the first author’s name, publication year, population country, total simple size, assay approaches and NOS score of each study were showed in Table 1. The information of adiponectin levels, age, BMI and AHI are given in Table 2.

**Table 1:** Characteristics of included studies

Author	Year	Country	N (OG/CG)	Adiponectin Source	Assay approach	NOS
Huang et al. [26]	2004	China	84 (69/15)	Serum	RIA	6
Zhang et al. [8]	2005	China	86 (66/20)	Serum	RIA	6
Wolk et al. [11]	2005	USA	55 (26/29)	Plasma	RIA	7
Sharma et al. [15]	2007	India	80 (40/40)	Plasma	ELISA	7
Ursavas et al. [12]	2010	Turkey	70 (55/15)	Serum	RIA	8
Ebru et al. [16]	2011	Turkey	50 (26/24)	Serum	RIA	7
Öztürk et al. [18]	2012	Turkey	94 (62/32)	Serum	ELISA	6
Sánchez et al. [17]	2012	Spain	41 (21/20)	Plasma	RIA	7
Kritikou et al. [19]	2013	USA	67 (36/31)	Serum	ELISA	7
Hargens et al. [10]	2013	USA	30 (12/18)	Serum	ELISA	8
Mutairi et al. [20]	2014	Kuwait	147 (105/42)	Plasma	ELISA	7
Xu et al. [28]	2014	China	82 (62/20)	Serum	ELISA	7
Wen et al. [27]	2015	China	48 (33/15)	Serum	ELISA	7
Kim et al. [22]	2015	Korea	59 (37/22)	Plasma	RIA	7
Araújo et al. [21]	2015	Brazil	53 (33/20)	Plasma	ELISA	7
Zuo et al. [29]	2016	China	89 (59/30)	Serum	ELISA	6
Lacedonia et al. [23]	2016	Italy	20 (10/10)	Serum	ELISA	7
Yang et al. [30]	2017	China	73 (35/38)	Serum	ELISA	7
Abdel et al. [24]	2017	Egypt	44 (22/22)	Serum	ELISA	7
Chen et al. [25]	2017	China	84 (69/15)	Serum	ELISA	8

*OG* OSAHS group, *CG* control group, *N* sample size, *NOS* Newcastle-Ottawa scale, *ELISA* Enzyme linked immunosorbent assay, *RIA* Radioimmunoassay

**Table 2:** Participants’ characteristics of included studies

Author	ADPN (Mean ± SD)		BMI (Mean ± SD), kg/m2		Age (Mean ± SD), y		AHI (Mean ± SD)	
	OG	CG	OG	CG	OG	CG	O G	CG
Huang et al. [26]	4.12 ± 2.49	7.74 ± 4.42	26.35 ± 2.60	26.02 ± 1.78	51.71 ± 12.37	56.37 ± 11.49	39.28 ± 22.80	3.67 ± 1.38
Zhang et al. [8]	4.23 ± 2.04	7.52 ± 2.21	26.8 ± 2.5	25.6 ± 1.8	50.7 ± 12.9	49.6 ± 9.2	36.8 ± 21.4	2.4 ± 1.8
Wolk et al. [11]	8.49 ± 4.69	6.32 ± 2.96	31 ± 5.10	31 ± 5.39	46 ± 5.10	46 ± 10.77	44 ± 20.40	3.0 ± 2.15
Sharma et al. [15]	4959.3 ± 3212.9	5706 ± 3670.8	29.8 ± 3.3	29.1 ± 2.3	42.3 ± 8.3	43.3 ± 7.8	32.19 ± 9.94	1.35 ± 0.61
Ursavas et al. [16]	7.7 ± 5.2	9.1 ± 6.6	32.5 ± 6.7	31.6 ± 7.0	51.1 ± 8.9	48.4 ± 11.6	43.5 ± 26.7	2.8 ± 1.5
Ebru et al.(mild) [16]	7.35 ± 2.7	14.87 ± 6.17	27.6 ± 1.8	28.4 ± 2.45	48 ± 27	47 ± 39.19	8.0 ± 2.52	2.05 ± 1.32
Ebru et al.(mod-sev) [16]	8.04 ± 6.55	14.87 ± 6.17	29.3 ± 4.12	28.4 ± 2.45	50 ± 27.11	47 ± 39.19	54.4 ± 66.79	2.05 ± 1.32
Öztürk et al.(mild) [18]	3.5 ± 4.2	5.2 ± 5.2	32.1 ± 6.6	31.3 ± 5.6	48.8 ± 10.6	48.3 ± 10.8	9.0 ± 2.6	1.8 ± 1.4
Öztürk et al.(mod) [18]	2.3 ± 1.2	5.2 ± 5.2	32.7 ± 5.8	31.3 ± 5.6	58.7 ± 8.6	48.3 ± 10.8	23.4 ± 4.6	1.8 ± 1.4
Öztürk et al.(sev) [18]	2.7 ± 3.2	5.2 ± 5.2	34.0 ± 5.5	31.3 ± 5.6	50.0 ± 11.7	48.3 ± 10.8	61.6 ± 20.4	1.8 ± 1.4
Sánchez et al. [17]	36.94 ± 21.42	29.47 ± 15.88	25.02 ± 1.22	24.71 ± 2.39	49.33 ± 10.71	42.9 ± 9.16	41.45 ± 18.3	2.87 ± 1.51
Kritikou et al.(male) [19]	4.75 ± 2.41	4.89 ± 2.55	27.09 ± 2.60	26.60 ± 2.65	53.87 ± 6.76	52.39 ± 6.23	42.42 ± 22.51	3.03 ± 1.98
Kritikou et al.(female) [19]	8.96 ± 5.48	11.63 ± 6.17	31.52 ± 1.54	30.36 ± 2.75	57.28 ± 6.00	54.21 ± 6.61	33.94 ± 18.78	1.69 ± 1.61
Hargens et al. [10]	10.0 ± 2.77	13.9 ± 5.94	32.4 ± 3.46	31.6 ± 4.67	22.8 ± 2.77	22.5 ± 2.97	25.4 ± 18.71	2.2 ± 1.27
Mutairi et al.(mild) [20]	10.53 ± 7.41	14.70 ± 13.01	43.3 ± 10.5	40.5 ± 11.9	49.1 ± 17.0	55.8 ± 16.3	10.3 ± 3.8	2.6 ± 1.6
Mutairi et al.(mod) [20]	10.64 ± 8.36	14.70 ± 13.01	49.8 ± 13.9	40.5 ± 11.9	53.0 ± 16.3	55.8 ± 16.3	18.0 ± 6.9	2.6 ± 1.6
Mutairi et al.(sev) [20]	8.87 ± 7.41	14.70 ± 13.01	44.4 ± 16.8	40.5 ± 11.9	50.0 ± 14.8	55.8 ± 16.3	48.9 ± 13.1	2.6 ± 1.6
Xu et al. [28]	7.98 ± 3.74	13.43 ± 2.04	–	–	50.8 ± 11.7	51.3 ± 10.5	18.03 ± 5.10	2.14 ± 0.89
Wen et al. [27]	6.13 ± 1.16	8.51 ± 3.84	27.4 ± 5.0	27 ± 4.6	48.5 ± 10.06	46 ± 10.4	41.3 ± 3.6	2.8 ± 0.4
Kim et al.(mod) [22]	8.08 ± 1.66	8.90 ± 2.63	24.43 ± 2.45	23.88 ± 2.30	38 ± 15.04	26 ± 6.91	14.40 ± 4.07	1.25 ± 1.25
Kim et al.(sev) [22]	6.88 ± 1.78	8.90 ± 2.63	28.69 ± 4.05	23.88 ± 2.30	42 ± 10.77	26 ± 6.91	52.71 ± 22.23	1.25 ± 1.25
Araújo et al. [21]	5.38 ± 0.44	5.98 ± 0.82	34.39 ± 0.51	34.51 ± 0.66	39.60 ± 1.48	32.50 ± 2.09	20.16 ± 3.57	2.55 ± 0.35
Zuo et al. [29]	6.4 ± 3.25	7.24 ± 2.42	27.3 ± 5.0	26.1 ± 1.7	47.3 ± 14.6	43.3 ± 7.8	20.6 ± 26.3	/
Lacedonia et al. [23]	63.43 ± 11.53	80.10 ± 18.50	27.29 ± 2.41	27.58 ± 1.40	62.80 ± 8.19	58.80 ± 17.55	/	/
Yang et al. [30]	8.13 ± 2.28	14.52 ± 4.10	24.61 ± 3.80	23.37 ± 3.55	50.12 ± 11.25	49.30 ± 10.70	37.87 ± 14.90	/
Abdel et al. [24]	5.09 ± 0.47	5.86 ± 0.89	36.00 ± 1.10	36.62 ± 1.14	49.92 ± 2.10	47.55 ± 2.35	32.17 ± 20.59	3.72 ± 1.69
Chen et al.(mod) [25]	29.31 ± 11.57	43.98 ± 22.13	25.35 ± 2.07	25.51 ± 2.17	43.32 ± 11.95	41.33 ± 12.45	32.12 ± 10.21	4.86 ± 2.31
Chen et al.(sev) [25]	25.79 ± 13.35	43.98 ± 22.13	26.12 ± 3.49	25.51 ± 2.17	42.06 ± 11.75	41.33 ± 12.45	72.47 ± 15.73	4.86 ± 2.31

*ADPN* adiponectin, *BMI* body mass index, *AHI* apnea-hypopnea index

### Pooled analysis

**Fig. 2:**
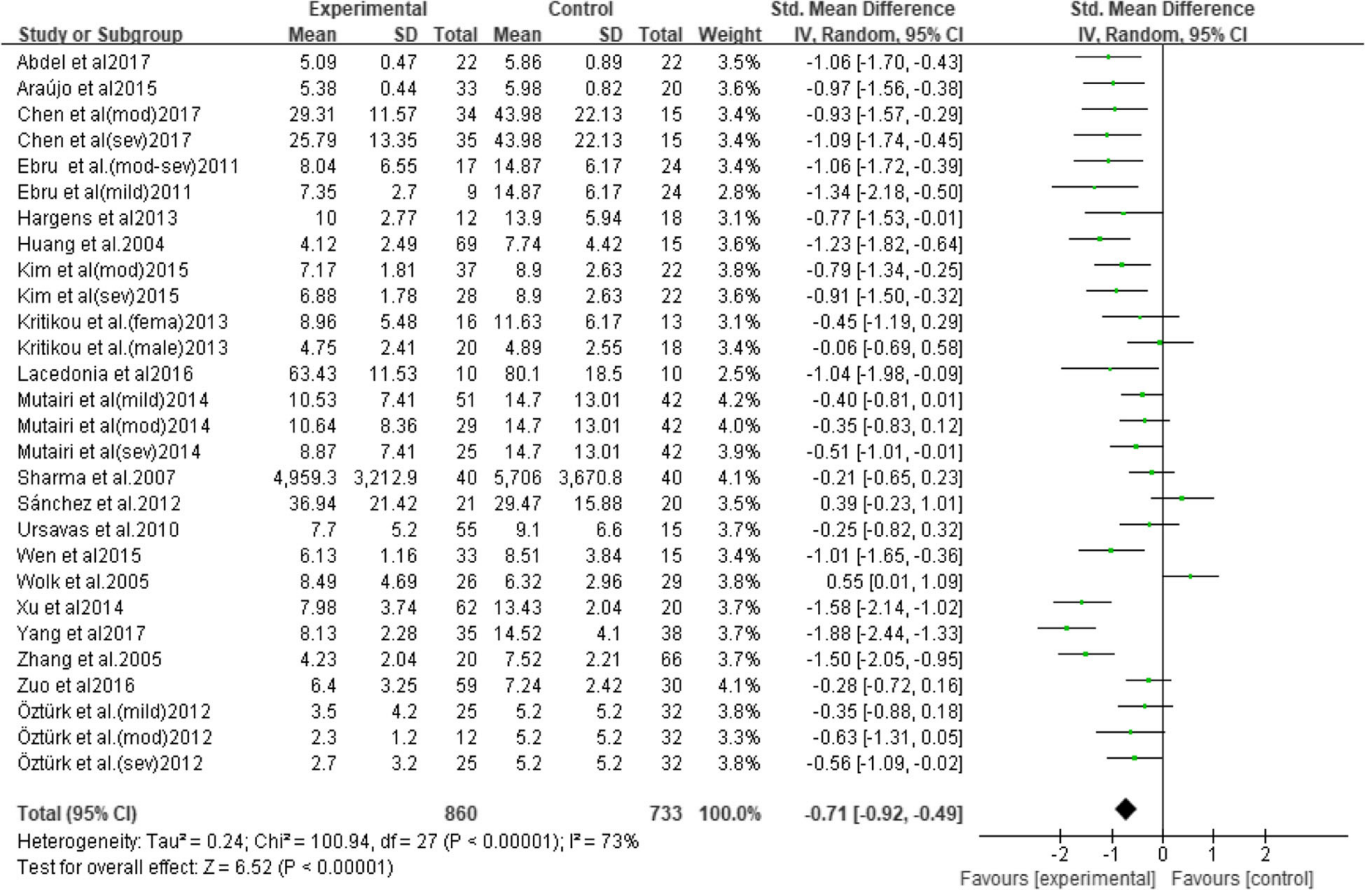
Forest plots of studies on adiponectin levels for OSAHS patients versus controls

The value of I^2^ was 73%, indicating that the studies were moderate heterogeneous. Therefore, the random effects model was used to combine effect size. Meta-analysis exhibited that serum/plasma adiponectin levels in OSAHS patients were significantly lower than that in controls (SMD = − 0.71, 95% CI = − 0.92 to − 0.49, *p* < 0.001) (Fig. 2).

### Subgroup analysis

Subgroup analysis stratified by race, adiponectin source, assay approaches, average age, BMI and AHI were performed, and the results were shown in Table 3. Results exhibited that adiponectin levels were significantly lower in OSAHS patients among all subgroup. In addition, we find that I^2^ would decrease when the subgroup analysis was stratified by race. Thus, race may be a potential source of heterogeneity.

**Table 3:** Subgroup analysis of adiponectin levels in osa patients and controls

Subgroup	N	SMD (95%CI)	z	p	Test of heterogeneity	
					I^2^	p
Overall	28	−0.71 (− 0.92, − 0.49)	6.52	< 0.001	73%	< 0.001
Race						
White	17	−0.42 (− 0.64, − 0.21)	3.87	< 0.001	56%	0.003
Nonwhite	11	−1.11 (−1.40, −0.82)	7.48	< 0.01	64%	0.002
Assay approaches						
ELISA	19	−0.72 (−0.94, − 0.50)	6.37	< 0.001	65%	< 0.001
RIA	9	−0.67 (−1.17, − 0.17)	2.63	0.009	84%	< 0.001
Adiponectin Source						
Serum	19	−0.89 (−1.13, − 0.65)	7.16	< 0.001	66%	< 0.001
Plasma	9	−0.36 (− 0.67, − 0.04)	2.20	0.03	71%	< 0.001
BMI						
BMI ≥ 30	12	−0.46 (−0.68, − 0.23)	3.89	< 0.001	49%	0.03
BMI < 30	15	−0.85 (−1.16, − 0.53)	5.25	< 0.001	76%	< 0.001
Age						
Age ≥ 50	13	−0.85 (−1.18, − 0.53)	5.15	< 0.001	74%	< 0.001
Age < 50	15	−0.58 (− 0.84, − 0.31)	4.25	< 0.001	69%	< 0.001
AHI						
AHI ≥ 30	17	−0.69 (−1.00, − 0.37)	4.29	< 0.001	80%	< 0.001
AHI < 30	10	−0.70 (− 0.97, − 0.43)	5.03	< 0.001	58%	0.01

### Sensitivity analysis

**Fig. 3:**
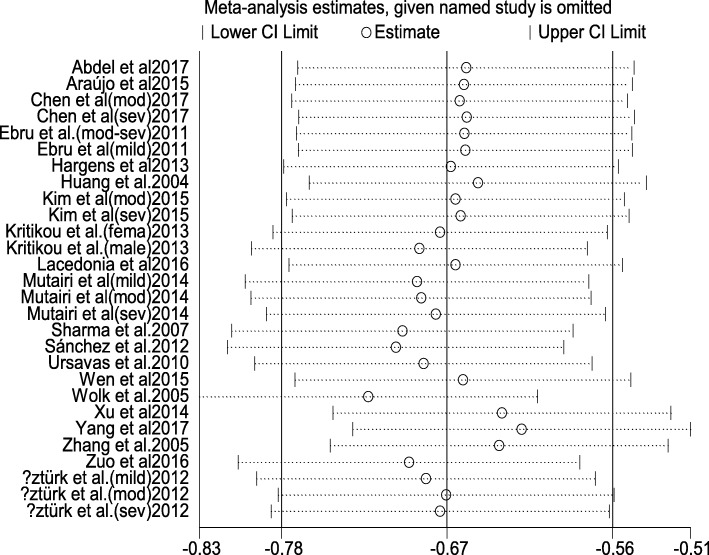
Sensitivity Analysis of studies on adiponectin levels for OSAHS patients versus controls

Sensitivity analysis were performed to assess the stability of the results (Fig. 3). The removal of any independent study did not significantly change the pooled results, suggesting these results were stable (data not shown). Pooled analysis using random-effect model showed that pooled SMD was − 0.71 (95% CI: -0.92, − 0.49), *P* < 0.001). The fixed-effect model drew a similar result which pooled SMD was − 0.66 (95% CI: -0.77 to − 0.55, *P* < 0.001).

### Publication Bias

**Fig. 4:**
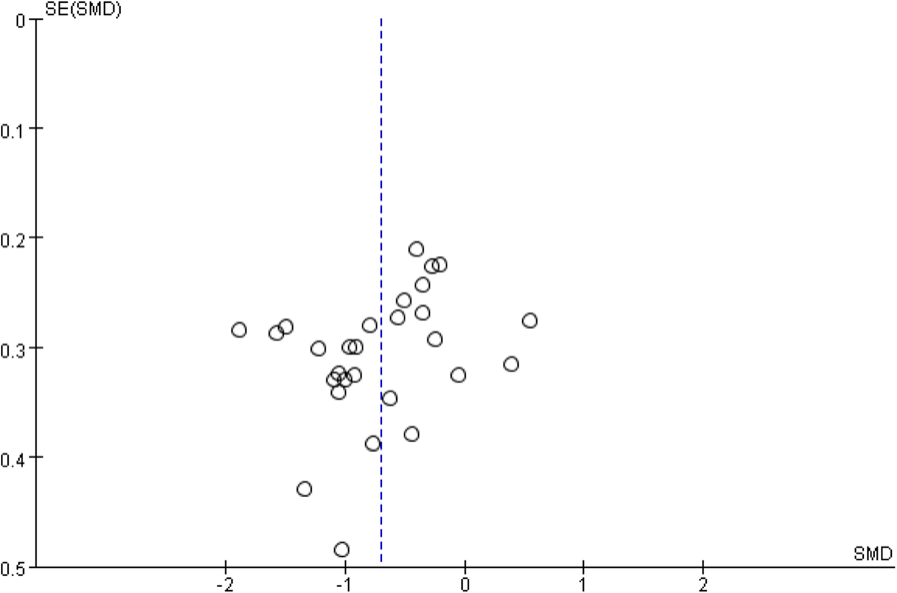
Funnel plot for all studies included in the meta-analysis

The funnel plot was not completely symmetrical, suggesting that the present study has some slight publication bias (Fig. 4). However, the Begg’s tests (*P* = 0.06) and Egger’s tests (*P* = 0.09) did not give sufficient evidence that the present study had publication bias.

### Meta-regression analysis

In meta-regression analysis, the outcome variable was the SMD of adiponectin level and the covariates included publication year, race, adiponectin source, assay approaches, average age, BMI and AHI. We can find that the adiponectin levels were significantly correlated with race (*p* = 0.002), but not significantly correlated with publication year (*P* = 0.56), adiponectin source(*P* = 0.09), assay approaches(*P* = 0.77), average age (*P* = 0.27), BMI (*P* = 0.12) and AHI (*P* = 0.28).

## Discussion

The current meta-analysis sought to summarize all available studies on the serum/plasma adiponectin levels among OSAHS patients and control subjects. We found that patients with OSAHS had significantly lower serum/plasma adiponectin levels compared with control subjects, indicating that adiponectin may play a role in the development of OSAHS. However, previous studies have found uncertain results for the association between adiponectin levels and OSAHS. Zhang et al. [8], Ebru et al. [16], Ozturk et al. [18] and Lacedonia et al. [23] have found OSAHS to be a potential driver of decreased adiponectin levels independent of age, gender and BMI. This was similar to our results. Hypoxia induced by OSAHS has been shown to reduce adiponectin levels via disruption of mechanisms that regulate the secretion of adiponectin. Moreover, other factors such as insulin resistance and hypoxia-induced sympathetic activation may also play significant roles in reducing adiponectin levels. While Sharma et al. in a cross-sectional [15] to determine whether obesity or OSAHS is responsible for adiponectin levels in patients with sleep disordered breathing, they found that no significant difference was noted in the OSAHS group compared to obese controls. Wolk et al. [11] reported that higher adiponectin levels in OSAHS patients compared to controls, which suggested that OSAHS may stimulate compensatory mechanisms, which can be considered to be protective of the cardiovascular system.

Moderate heterogeneity was observed among these studies. Therefore, subgroup analysis and meta-regression analysis were performed to detect the potential source of heterogeneity. In subgroup analysis, only stratification by race I^2^ would result in a decrease of I^2^ in both white and non-white groups. In addition, meta-regression analysis also suggested that the adiponectin levels were only significantly related with race. We can speculate race may be the potential source of heterogeneity in this present meta-analysis. Therefore, the relationship between serum/plasma adiponectin levels and OSAHS from different races requires further investigation and especially the exact composition of the non-white group.

To our knowledge, this is the first meta-analysis conducted to assess the relationship between serum/plasma adiponectin levels in OSAHS patients and in control subjects. Most of clinical research studies have focused primarily on older obese males with apnea because OSAHS is more common in males and in overweight/obese populations [31, 32]. However, in these studies the findings were confounded by obesity, gender and age. To address these confounded factors, we firstly excluded the impact of age, gender and BMI on serum/plasma adiponectin levels in our meta-analysis. Hence, a major strength of our meta-analysis is that no statistically significant difference was found between OSAHS patients and controls in terms of age, gender and BMI. Another strength of this meta-analysis is the large sample size among patients with OSAHS. Moreover, none of the patients included had obvious coronary heart disease or chronic respiratory diseases. Performing a subgroup analysis and meta-regression analysis to better understand the effect of race, adiponectin source, assay approaches, average age, BMI and AHI on the serum/plasma adiponectin levels is also another strength of this study. However, several limitations should be acknowledged. Even though we used a broad search strategy, we cannot claim to have been exhaustive in retrieving all studies. In addition, some nonsignificant findings from existing studies remain unpublished, which may have some important information. We also found some conference abstracts that might have been included, but they lacked substantial details on some important data. A major limitation of the present study is a new bias may be introduced because of the confounding factor adjustment, which would affect the accuracy of the combined effect value. Finally, meta-analysis remains retrospective research, which is impossible to avoid the methodological deficiencies of the included studies. Therefore, the relationship between serum/plasma adiponectin levels and OSAHS needs to be verified given the limited number of studies.

In conclusion, the serum/plasma adiponectin levels were significantly lower in OSAHS patients than that in control subjects, suggesting a potential role of adiponectin in OSAHS pathogenesis, deserves further studies as a potential marker of OSAHS.

## Abbreviations

ADPN: Adiponectin
AHI: Apnea hypopnea index
BMI: Body mass index
CI: Confidence interval
ELISA: Enzyme linked immunosorbent assay
IR: Insulin resistance
NOS: Newcastle-Ottawa scale
OSAHS: Obstructive sleep apnea hypopnea syndrome
RIA: Radioimmunoassay
SMD: Standard mean difference
